# Magnesium isoglycyrrhizinate inhibits airway inflammation in rats with chronic obstructive pulmonary disease

**DOI:** 10.1186/s12890-021-01745-7

**Published:** 2021-11-15

**Authors:** Ye Yang, Lei Huang, Chongchong Tian, Bingjun Qian

**Affiliations:** Department of Pharmacology and Medicinal Chemistry, Jiangsu Vocational College of Medicine, Yancheng, 224005 Jiangsu People’s Republic of China

**Keywords:** MgIG, COPD, IL-6, TNF-α, NLRP3 inflammasome

## Abstract

**Background:**

Chronic obstructive pulmonary disease (COPD) is a kind of chronic lung diseases with the characteristics of airway remodeling and airflow obstruction. Magnesium isoglycyrrhizinate (MgIG) is an anti-inflammatory glycyrrhizic acid preparation for treating hepatitis. However, whether MgIG can treat other diseases and its action mechanism is still obscure. In this study, we evaluated the anti-inflammatory effect of MgIG in rats with COPD and investigated the underlying mechanisms.

**Methods:**

Rat model of COPD was constructed by endotracheal-atomized lipopolysaccharide exposure and cigarette smoke induction. Rats were randomly divided into 5 groups: control group, COPD model group, salmeterol fluticasone comparator group, low dose of MgIG group, and high dose of MgIG group. Except for normal control group, the other four groups received sensitization treatment by cigarette smoking and endotracheal-atomization of endotoxin lipopolysaccharide to construct COPD rats model. After model established successfully, the COPD rats in each group received corresponding dose of endotracheal-atomized normal saline, salmeterol fluticasone, and MgIG every day prior to exposure of cigarette smoke from days 30 to 45. Normal control group were treated with normal saline. Finally, All rats were euthanatized. Pulmonary function was measured. Cells in bronchoalveolar lavage fluid were classified, inflammatory factors IL-6 and TNF-α were determined, histopathological analysis was performed by HE staining, and expression of NLRP3 and cleaved caspase-1 in the lung tissue was also determined by Western blotting.

**Results:**

It showed that MgIG treatment (0.40 or 0.80 mg/kg/day) could recover the weight and the clinical symptoms of rats with COPD, accompanied with lung inflammation infiltration reduction, airway wall attenuation, bronchial mucus secretion reduction. Additionally, MgIG administration reduced inflammatory cells (white blood cells, neutrophils, lymphocytes and monocytes) accumulation in bronchoalveolar lavage fluid and decreased IL-6 and TNF-α production in the serum of COPD rats. Furthermore, MgIG treatment also reduced the expression level of NLRP3 and cleaved caspase-1.

**Conclusion:**

It indicate that MgIG might be an alternative for COPD treatment, and its mechanism of action might be related to the suppression of NLRP3 inflammasome.

**Supplementary Information:**

The online version contains supplementary material available at 10.1186/s12890-021-01745-7.

## Background

Chronic obstructive pulmonary disease (COPD) is a kind of chronic lung disease characterized by persistent airflow obstruction caused by lung structure destruction, mucus block in airway, and inflammation and swelling of the airway lining, which can further develop into common chronic diseases of pulmonary heart disease and respiratory failure [1, 2]. COPD is commonly associated with abnormal inflammatory response of harmful gases and granules accompanied with high disability and mortality [3]. At present, the age-standardized incidence rate of COPD for both sexes combined in 2017 has reached 0.9‰ to 5.03‰ across the world, and the overall incidence rate of COPD in the adult populations aged over 45, living in Ommoord (a suburb of the city of Rotterdam, the Netherlands), even reached to about 9‰ person-years [4, 5]. In China, the mortality of COPD ranks the third among all causes of death, seriously affecting patients’ life quality and aggravating the social and economic burden [6]. Therefore, new therapeutic strategies and drugs are needed to prevent airway inflammation in COPD.

It has been generally recognized that the central feature of COPD is inflammation, which causes the pathological changes in all different parts of the lung [7–10]. Lung inflammation can cause a considerable increase in leukocytes, lymphocytes (especially CD8^+^ T cells), neutrophils in different lung compartments [11]. Activated inflammatory cells release a variety of mediators, such as proinflammatory cytokines: leukotriene B4 (LTB4), interleukin-6 (IL-6), interleukin-8 (IL-8), interleukin-1β (IL-1β), tumor necrosis factor alpha (TNF-α) and other inflammatory mediators [12], contributing to the lung structural damage and/or neutrophil inflammatory response exaggeration. And, the serum IL-6 and TNF-α level can reflected the severity of COPD, indicating that they can be used as biomarkers of the systemic inflammatory response in COPD patients [13]. So, chronic inflammation of airway and lung is the main driver of the occurrence and development of COPD [14]. However, the mechanisms involved in the production and massive spread of inflammation are not clear. Inflammasomes play an important role in the occurrence and development of inflammation in respiratory diseases [15]. Cigarette smoke could cause the activation of the nucleotide-binding oligomerization domain-like receptor protein 3 (NLRP3) inflammasomes, resulting in an increase in IL-1β and IL-18 [16]. So, NLRP3 might play an important role in chronic inflammation of COPD.

**Fig. 1:**
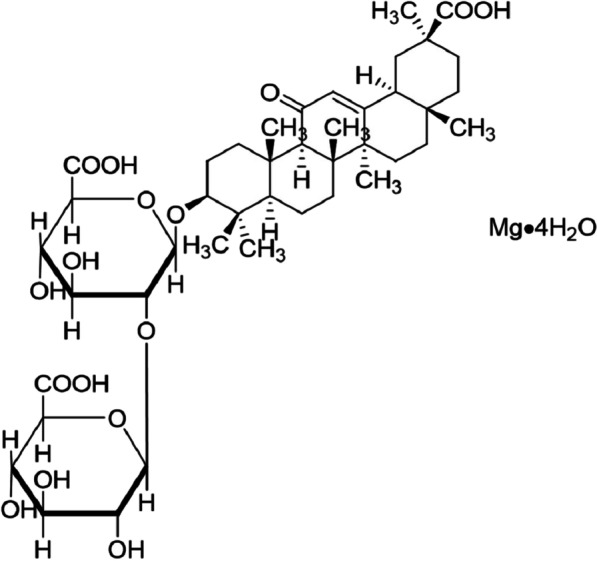
Chemical structure of MgIG. Systematic name, (18α, 20β)-carboxy-11-oxo-oleanorane-12-en-3β-yl-2-O-β-d-glucopyranosiduronyl-α-d-glucopyranosiduronic acid Magnesium Tetrahydrate; formula,C_42_H_60_MgO_16_·4H_2_O

As COPD severity increases, all existing drugs get limited to a palliative role, such as corticosteroids and bronchodilators, and therapies with them are associated with side effects [17, 18]. Some natural molecules, such as flavonoids and polyphenols, also exhibit anti-inflammatory activity. Licorice (*Glycyrrhiza glabra*) is one of the most widely used herbs in traditional Chinese medicine (TCM) for treating asthma, pharyngitis and infections due to its antiviral and antimicrobial, anti-inflammatory, immunomodulatory, and antitussive and expectorant pharmacological actions for centuries [19], whose main functional chemicals is glycyrrhizic acid (also named glycyrrhizin) concentrated in the root. And, doses of glycyrrhizic acid below 100 mg/day could prevent side effects [19]. Based on this, glycyrrhizic acid preparations or derivatives, such as magnesium isoglycyrrhizinate (MgIG, Fig. 1), have been developed and applied in clinical treatment of inflammatory diseases [20–23]. Previous studies have confirmed the therapeutic effect of MgIG in rats on lung injury caused by herbicides [21]. In particular, it has been recently reported that clinical application characteristics of MgIG suggests it as a potential adjuvant treatment in the "Management Standard for Mild and Common Patients of Coronavirus Disease 2019 (COVID-19) (Second Edition)", issued by National Health Commission of People's Republic of China and National Administration of Traditional Chinese Medicine [24]. Naturally, it could be hypothesized that MgIG could alleviate the inflammatory response in lungs during COPD. The objective of this study was to evaluate the anti-inflammation effect of MgIG in lungs of COPD model rats and explore the underlying mechanisms.

## Materials and methods

### Preparation of MgIG

MgIG injection was purchased from Chia Tai Tianqing Pharmaceutical Group Co., Ltd. (Lianyungang, China). The main chemical composition of this product is MgIG, with the molecular formula of C_42_H_60_MgO_16_·4H_2_O. All drugs were stored at 4 °C prior to use.

### Rat model of COPD and MgIG treatment.

The male Wistar rats (body weight, 200 ± 20 g; age, 8–10 weeks) were purchased from the Shanghai Super-B&K Laboratory Animal Co., Ltd. (Shanghai, China). The rats were housed in cages and allowed ad libitum access to rat standard diet and water throughout the experiment under the specific pathogen-free conditions with room temperature of 21–23 °C, humidity of 60 ± 5%, and a 12 h light/dark cycle.

**Fig. 2:**
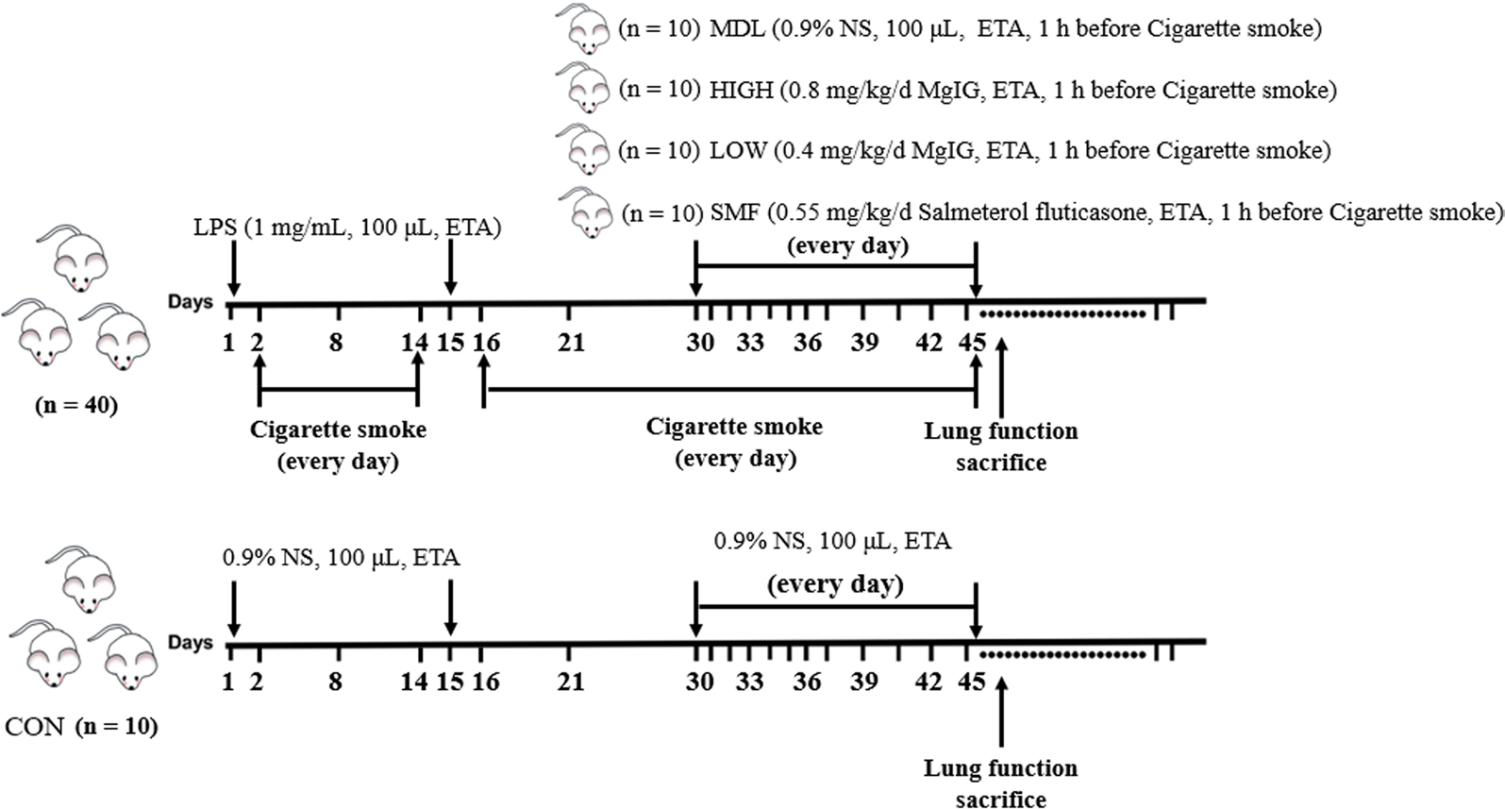
A flowchart for the establishment of COPD rat model and administration of MgIG. Abbreviations: ETA, endotracheal atomization

The COPD model in rats was established by cigarette smoking and endotracheal-atomization (ETA) of endotoxin lipopolysaccharide (LPS) shown in Fig. 2, according to a previous report [25]. Rats were randomly divided into 5 groups (10 rats in each group): control group (CON), COPD model group (MDL), Salmeterol Fluticasone comparator group (SMF), low dose of MgIG group (LOW), High dose of MgIG group (HIGH). Rats were anesthetized with isoflurane gas on days 1 and 15 and subsequently sensitized by an endotracheal-atomization with 100 μL of 1 mg/mL LPS using liquid aerosol devices (MicroSprayer® Aerosolizer, Model IA-1B, Penn-Century, Inc. Wyndmoor, USA), respectively, and rats were also placed in several fumigation boxes and passively exposed to 5% (v/v) cigarette smoke produced by Hademen cigarettes (Jinan, China), each of which contains 10 mg tar, 1.0 mg nicotine content, and 12 mg carbon monoxide, for 30 min twice with an interval of 6 h daily from days 2 to 14, except for CON group. After the COPD model rat was established successfully, rats were challenged by cigarette smoke alone with the method aforementioned every day from days 16 to 45. Additionally, SMF group received 0.55 mg/kg/d (w/bw/days) salmeterol fluticasone dissolved in normal saline, LOW group received 0.40 mg/kg/d (w/bw/days) MgIG, and High group received 0.80 mg/kg/d (w/bw/days) MgIG by endotracheal-atomization 1 h before cigarette smoke treatment from days 30 to 45, respectively, whereas MDL group received 100 μL of 0.9% normal saline with the same delivery method. Rats in CON group were sensitized and challenged with 100 μL of normal saline in the same way and exposed to the normal air. After 45 days, rats were sacrificed with an intraperitoneal injection of pentobarbital sodium (150 mg/kg; Sigma-Aldrich; Merck KGaA). All experimental procedures were executed in accordance with the Guidelines and Regulations for the Use and Care of Animals of the Center for Laboratory Animal Care of Jiangsu Vocational College of Medicine and approved by Ethics Committee of Jiangsu Vocational College of Medicine. All methods were conducted in accordance with the ARRIVE guidelines. (https://arriveguidelines.org).

### Pulmonary function measurement

The pulmonary function of the rats was evaluated with the AniRes2005 lung function analysis system (Beilanbo Technology Co., Ltd, Beijing, China), according to the manufacture’s instructions. Briefly, at the end of 45-day treatment, rats were anesthetized using 3% pentobarbital sodium (70 mg/kg; Sigma-Aldrich; Merck KGaA) and placed into a volume box in the fixed supine position. Subsequently, endotracheal intubation was executed on the rats. The intubation tube was connected to the ventilator and signal conditioner, transmitting the data of air flow and change in volume trancing box to the computer. The respiratory rate and time ratio of expiration/inspiration were preset at 75/min and 1.5:1, respectively. The forced vital capacity (FVC) and forced expiratory volume in 0.3 seconds (FEV0.3) were detected, and the ratio of the two (FEV0.3/FVC) was used as the index to evaluate the lung function of rats.

### Cells classification in bronchoalveolar lavage fluid (BALF).

To measure airway inflammation, the inflammatory cells accumulated in bronchoalveolar lavage fluid (BALF) were analyzed. Following anesthetizing rats, tracheas were surgically exposed and intubated. BALF was harvested by lavage with 3 sequential 1 mL of Hank's balanced salt solution without calcium and magnesium. The collected lavage fluid was placed on ice, and then centrifuged (400 g) at 4 °C for 10 min. The cell pellet was resuspended in 1 mL of Hank's balanced salt solution, and the total number of cells in BALF was counted with a standard haemocytometer. Smears were prepared by cell centrifugation, stained with Liu's staining solution (PS0290, Beijing Jin Ming Biotechnology Co., Ltd. China). According to the standard morphological criteria, approximately 200 cells were counted and classified as macrophages, lymphocytes, neutrophils, and eosinophils.

### Measurement of inflammatory factors

To evaluate the effect of MgIG on the production of proinflammatory cytokines, the serum of cigarette and LPS induced COPD rat were collected and diluted in 1:20 for detection. The levels of inflammatory factors, interleukin (IL)-6 and tumor necrosis factor (TNF)-α, were determined with ELISA kits MBS7625314 and RTA02 from MyBioSource (San Diego, CA, USA) according to the manufacturer's protocol.

### Histopathological analysis

In order to evaluate the effect of MgIG treatment on cigarette smoke combined with LPS-induced COPD, histopathological examination was performed in each group according to the method described previously [26]. The left upper lobe of each group was removed, rinsed with ice-cold normal saline, and fixed in 10% formalin (Shanghai Jianxin Chemical Co., Ltd., Shanghai, China). The tissues were sliced, embedded in paraffin (Shanghai Yongye Biological Technology Co., Ltd., Shanghai, China), and prepared into 4 µm-thick sections using a Leica RM 2135 microtome (Leica Microsystems GmbH, Wetzlar, Germany) for histological analysis.

The sections were stained with haematoxylin and eosin (HE) for observation under a light microscope. The mean alveolar number (MAN), and bronchial wall thickness were determined. Cell infiltration was scored as follows: 0, no cells; 1, a few cells; 2, a ring of cells and 1 cell deep; 3, a ring of cells and 2–4 cells deep; and 4, a ring of cells and > 4 cells deep. The scores of ten rats were averaged. The sections were observed independently by three pathologists in a blinded manner.

### Western Blotting

The levels of NLRP3 and cleaved caspase-1 in rat was detected by western blotting. The lung tissue homogenates were prepared in lysis buffer (Beyotime, Shanghai, China), containing 1 nM phenylmethanesulfonyl fluoride (PMSF) (Beyotime, Shanghai, China), and proteins were obtained after centrifugation (80009, 10 min). The concentrations of proteins were determined using a BCA protein assay kit (Beyotime, Shanghai, China), and 300 μg proteins from each sample were then separated using 10% SDS-PAGE. After transferring to PVDF membranes, the membranes were blocked with a 5% BSA solution for 1 h and incubated overnight with primary antibodies anti-NLRP3 (EPR23073-1, 1:1000 Abcam, Cambridge, United Kingdom) and anti-caspase 1 (ab138483, 1:1000, Abcam, Cambridge, United Kingdom). Then membranes were incubated continually with HRP-conjugated secondary antibody (1:10,000 dilution in TBST containing 5% skimmed milk), and the bands were imaged using a Western blot detection kit (Beyotime, Shanghai, China). The blot was scanned and analyzed using UN-SCAN-IT version 5.1 software to measure the band densitometry. Original western blotting gels were provided in Additional file 1.

### Statistical analysis

All data are presented as the mean ± standard deviation, and analyses were performed with Graphpad Prism 8 software. Comparisons between experimental groups were conducted using Tukey's after ANOVA for group to group comparison, Values of *P* < 0.05 were considered significant.

## Results

### Effect of MgIG on body weight, clinical symptoms and pulmonary functionin of rats with COPD.

**Fig. 3:**
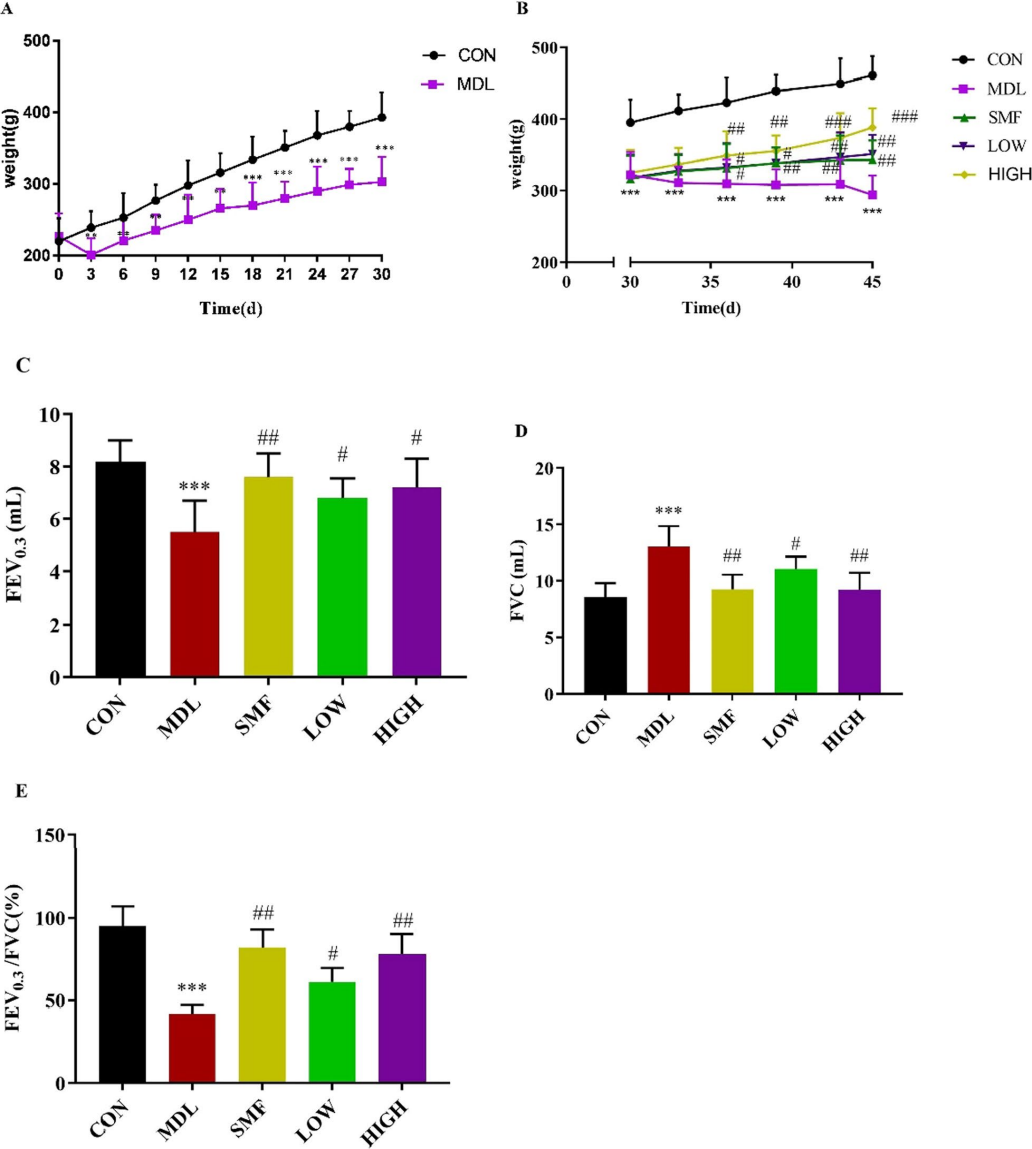
Effects of MgIG on body weight and pulmonary function in COPD rats. **A** Changes of body weight of the rats during 30-day COPD model construction. **B** Changes of body weight of the rats in different groups during treatment. **C** Comparison of FEV0.3 of rats in different groups after treatment for pulmonary function evaluation. **D** Comparison of FVC of rats in different groups after treatment for pulmonary function evaluation. E, Comparison of FEV0.3/FVC of rats in different groups after treatment for pulmonary function evaluation. All data were presented as mean ± standard deviation (n = 10 in each group). Statistical significance of differences were determined by one-way ANOVA. ***P < 0.001 and **P < 0.01 compared to CON group. ^###^*P* < 0.001, ^##^*P* < 0.01 and ^#^*P* < 0.05 compared to MDL group. Abbreviations: FEV0.3, forced expiratory volume in 0.3 s; FEV0.3/FVC, forced expiratory volume in 0.3 seconds/forced vital capacity; FVC, forced vital capacity

The weight of rats in MDL group decreased compared with that in CON groups during 30 days of COPD rat model construction (*P* < 0.01, Fig. 3A). Whereas, the subsequent treatment with the low and high dose MgIG could significantly recover the growth of these rats, same as treatment with salmeterol fluticasone (*P* < 0.05 or *P* < 0.01, Fig. 3B). Compared with CON group, FEV_0.3_/FVC in MDL group decreased significantly (*P* < 0.01), which was reversed in the SMF, LOW and HIGH group significantly (Fig. 3C–E). These data suggest that 0.80 mg/kg MgIG treatment have therapeutic effects in rat with COPD.

### MgIG attenuates lung inflammatory responses in a rat model of COPD

**Fig. 4:**
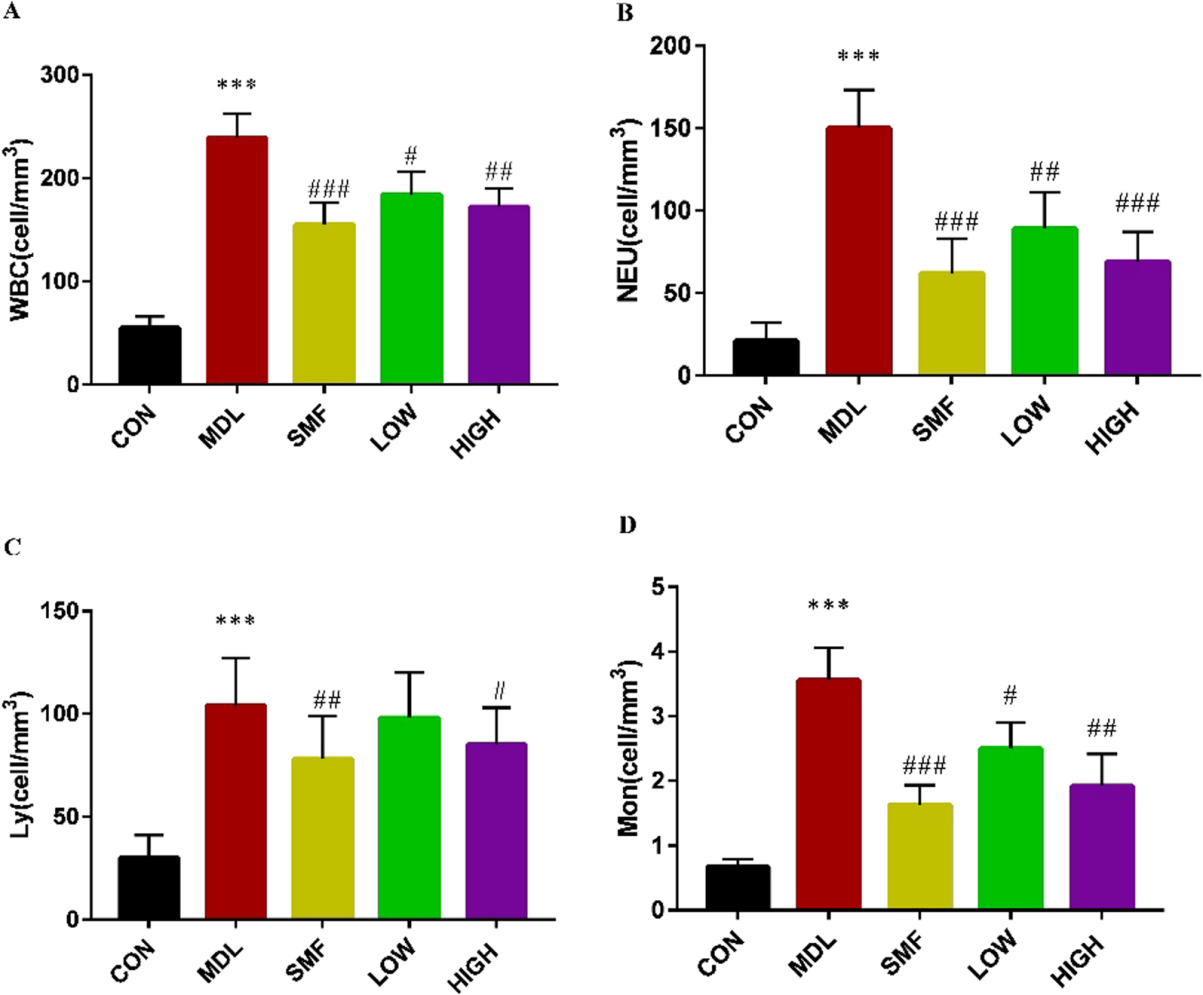
Effects of MgIG on the inflammatory cells proliferation in BALF of COPD rats. A, Number of white blood cells in the BALF of rats after 15-day different treatment. B, Number of neutrophils in BALF of rats after 15-day different treatment. C, Number of lymphocytes in BALF of rats after 15-day different treatment. D, Number of monocytes in BALF of rats after 15-day different treatment. All data were presented as mean ± standard deviation (n = 10 in each group). Statistical significance of differences were determined by one-way ANOVA. ***P < 0.001 compared to CON group. ^###^*P* < 0.001, ^##^*P* < 0.01 and ^#^*P* < 0.05 compared to MDL group

Inflammation plays an important role in the development of COPD. We evaluated the effect of MgIG treatment on it. Compared with CON group, the number of leukocytes, neutrophils and lymphocytes in BALF in MDL group was significantly increased. And, high and low dose of MgIG treatment significantly reversed leukocytes, neutrophils and Monocytes accumulated in BALF of the COPD rats (*P* < 0.05 or *P* < 0.01, Fig. 4A–D). The 0.80 mg/kg MgIG treatment reduced the number of lymphocytes significantly as 0.55 mg/kg salmeterol fluticasone treatment, but 0.40 mg/kg MgIG treatment did not (Fig. 4C).

### MgIG reduced proinflammatory cytokines expression in the serum of COPD rat

**Fig. 5:**
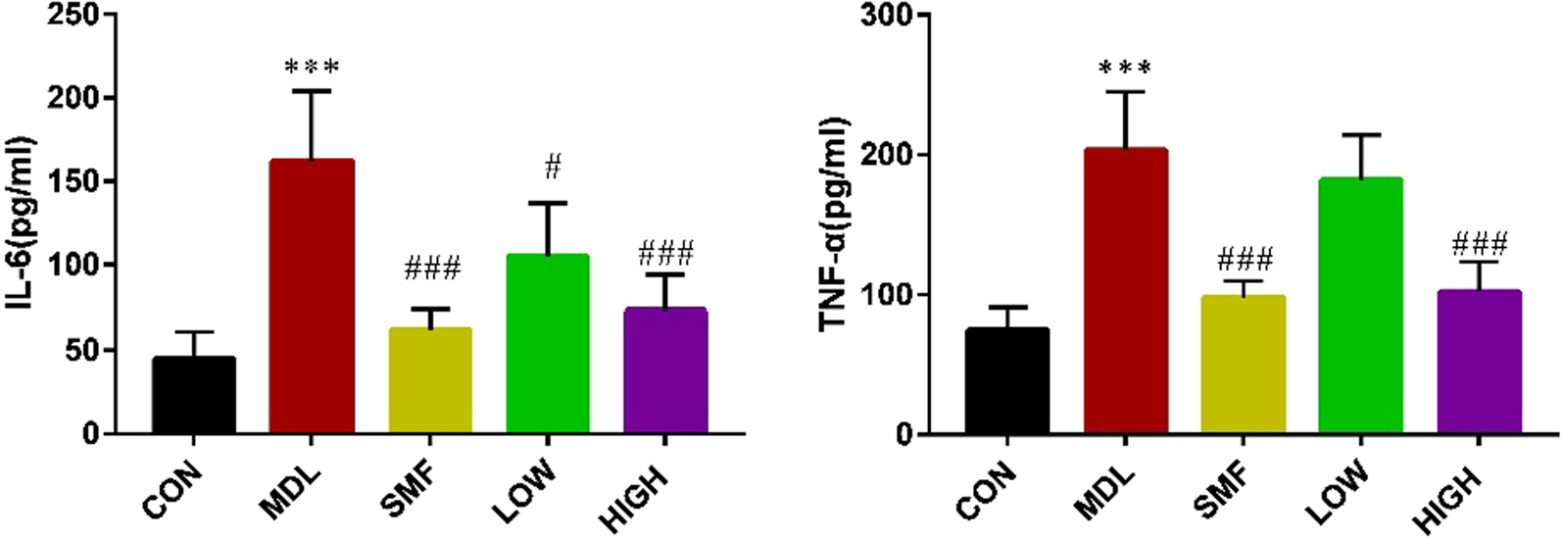
Effect of MgIG on the proinflammatory cytokines in the serum of COPD rats. Rats were sacrificed and the serum was collected for ELISA detection. The levels of IL-6 (**A**) and TNF-α (**B**) were shown as above. All data were presented as mean ± standard deviation (n = 10 in each group). Statistical significance of differences were determined by one-way ANOVA. ***P < 0.001 compared to CON group. ^###^*P* < 0.001, ^##^*P* < 0.01 and ^#^*P* < 0.05 compared to MDL group

IL-6 and TNF-α are important inflammatory factors and play an important role in the progression of COPD. The levels of IL-6 and TNF-α in the serum of MDL group were significantly higher than those in CON group. In contrast, the expression of IL-6 and TNF-α were significantly suppressed by MgIG treatment (*P* < 0.05 or *P* < 0.01, Fig. 5A, B), exhibiting dose–effect relationship. It suggested that MgIG could suppress inflammation development caused by COPD.

### Effect of MgIG on histopathology in rats with COPD

**Fig. 6:**
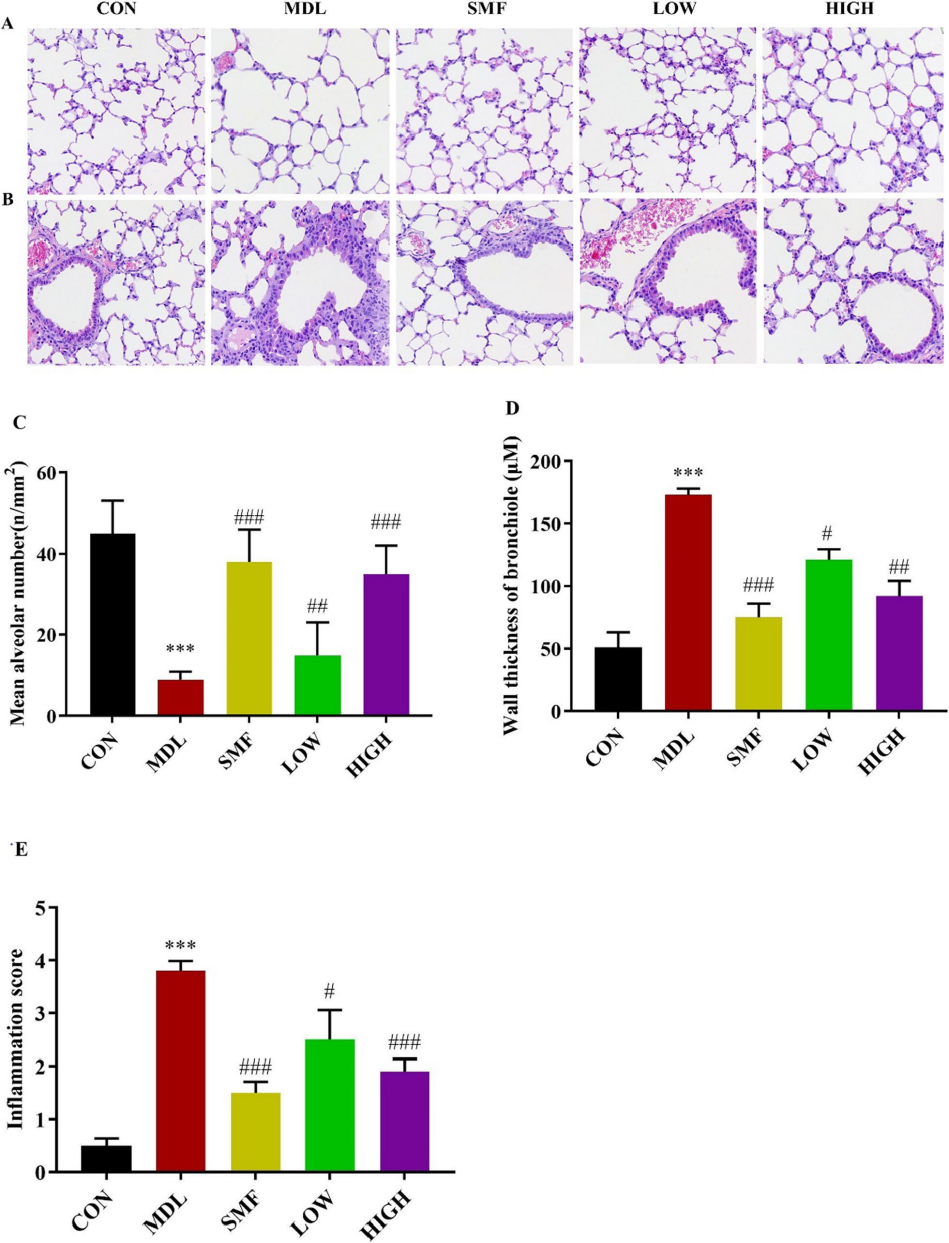
Effect of MgIG on histopathology in rats with COPD. **A** the images show the alveoi (HE × 400). **B** the images show the airway (HE × 400). **C**–**E** Mean alveolar volume, bronchial wall thickness and inflammation score were measured to compare the effect of MgIG in rats with COPD. All data were presented as mean ± standard deviation. Statistical significance of differences were determined by one-way ANOVA. ***P < 0.001 compared to Control group. ^###^*P* < 0.001, ^##^*P* < 0.01 and ^#^*P* < 0.05 compared to Model group

In order to detect the pathological changes of airway structure in COPD rats, the sections of each group were stained with HE. As demonstrated in Fig. 6A, B, compared to the control group, the lungs of rats in the MDL group exhibited inflammatory cell infiltration, bronchial wall thickening and mucous plug formation, as well as expansion of the alveolar volume (Fig. 6A, B). After MgIG intervention, rats in the LOW and HIGH group exhibited some degree of improvement in pathological changes, including inflammatory cell infiltration reduction, bronchial wall thinning, mean alveoli number decrease, and alveolar dilatation and rupture alleviation (*P* < 0.05 or *P* < 0.01, Fig. 6C–E).

### MgIG suppresses the expression of NLRP3 inflammasome in COPD rats induced by cigarette and LPS

**Fig. 7:**
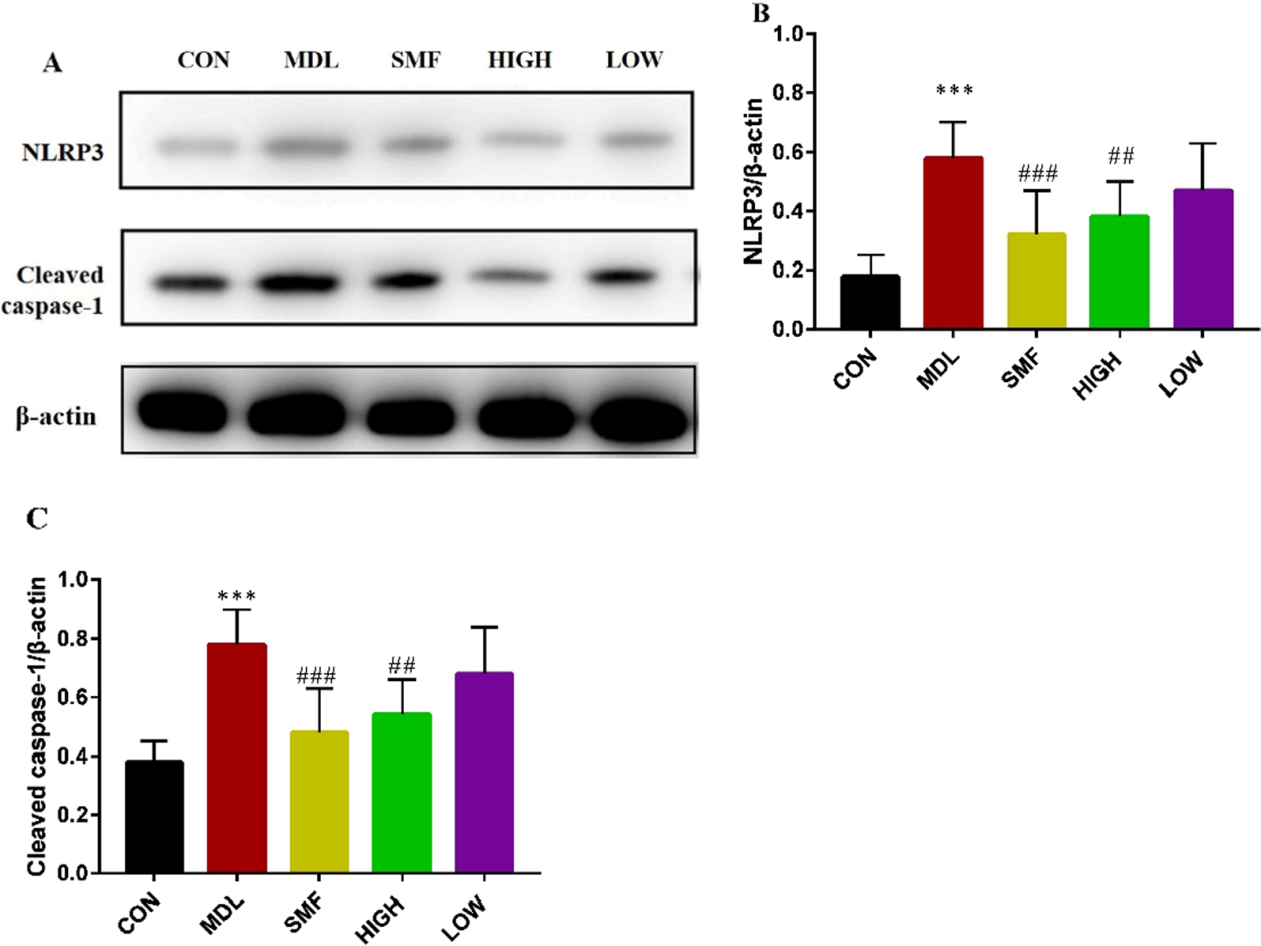
Effect of MgIG on the expression of NLRP3 inflammasome and cleaved caspase-1 in COPD rats. After the rats were sacrificed, the upper lobe of the right lung was removed for western blotting. **A** The protein levels of NLRP3 and cleaved Caspase-1 were detected by western blotting, and the ratio of NLRP3/β-actin (**B**) and cleaved Caspase-1/β-actin (**C**) were shown. All data were presented as mean ± standard deviation (n = 10 in each group). Statistical significance of differences were determined by one-way ANOVA. ***P < 0.001 compared to CON group. ^###^*P* < 0.001 and ^#^*P* < 0.05 compared to MDL group

Western blotting analysis revealed that the expression levels of NLRP3 and cleaved caspase-1 was dramatically decreased in lung tissue of rats in LOW and HIGH groups compared to those in MDL group (*P* < 0.05 or *P* < 0.01, Fig. 7A–C).

## Discussion

COPD is a common chronic lung disease which can cause some respiratory symptoms, including cough, breathlessness, and excessive phlegm. To investigate the pathophysiologic mechanism and explore potential drugs for COPD, many animal COPD models were established, among of which cigarette smoke induced rat COPD model is commonly applied [27]. Considering difficulty in capturing both chronic bronchitis-related or emphysematous changes in a single model owing to complex COPD pathophysiology, we used LPS combined with cigarette smoke as stimuli to construct rat COPD model. The analysis result of histopathology confirmed that bronchial mucus secretion, inflammatory cells, and airway wall thickness increased in rats from MDL group.

MgIG is a new drug for liver protection, which is obtained from glycyrrhizic acid extracted from natural plant *Glycyrrhiza uralensis* by alkali catalysis [28] and isomerization. *Glycyrrhiza uralensis* has been used in TCM to treat lung disease for centuries due to its pharmacological and therapeutic effects [29–31]. The present study investigated the suppression effect of MgIG on airway inflammatory in rats with COPD. Previous studies had indicated that long-term and/or large amounts use of glycyrrhizic acid could cause side effects, such as peseudohyperaldosteronism and hypertension, due to its hyper-mineralocorticoid-like effect. So, it is important to choose safety dosage according to mode of administration and receptor species. The intraperitoneal dose of MgIG was 100 mg/kg/day in mice [32], 15–45 mg/kg/day in rats [21], and the oral dose of MgIG was 15 mg/kg in rats [33], which all did not cause side effect. The mean bioavailability of glycyrrhizin in rats after intraperitoneal administration was about 80.0% [34], and the maximum observed concentration (C_max_) of MgIG in plasma was about 99.28 mg/L in health human weighed between 50.8 and 86.7 kg after intravenous administration with dosage of 300 mg dissolved in 250 mL 5% glucose [35]. Taking these information together, we chose 0.4–0.8 mg/kg/day as endotracheal-atomization dosage in this study, and the results indicated that MgIG can alleviate the pathological changes in COPD rats, but not cause other untoward effect.

Complex airway inflammation can cause airway obstruction and progressive airway remodeling in COPD rats [36, 37]. Various inflammatory factors and proinflammatory cytokines are involved in airway injury [38]. Previous studies demonstrated that the serum IL-6 and TNF-α level were the key critical biomarker of COPD, which were closely correlated with the severity degree of COPD [13]. Additionally, neutrophils enter the lungs and are activated under the action of chemokines, further aggravating the inflammatory response [39]. MgIG has been indicated to have effects on anti-inflammatory and immunomodulatory activities in vitro and in vivo [22, 23]. In the present study, MgIG can suppress the production of proinflammatory factors TNF-α and IL-6 in the serum of COPD rats, and the increase of white blood cells, neutrophils and lymphocytes in BALF of rats in MDL group could be significantly counteracted by MgIG treatment. It means that MgIG has anti-inflammatory activity in vivo. Overall, the current results support that inhibition of airway inflammation may contribute to the protective effect of MgIG on COPD.

A few studies have revealed that NLRP3 inflammasome is related to a series of chronic inflammatory diseases [40, 41]. Inflammasomes can recognize pathogen-associated molecular patterns (PAMPs), and then recruit and activate proinflammatory protease caspase-1, which can further cleave IL-1 β, IL-18 precursor contributing an increased secretion of proinflammatory cytokines IL-1 β and IL-18. In addition, the activation of inflammasomes can also induce cell inflammatory necrosis [42]. In this study, the relative expression of NLRP3 and cleaved caspase-1 protein were significantly increased in the lung tissues of rats in MDL group (*P* < 0.05). It suggests that the mechanism of COPD may be due to the activation of NLRP3 and Cleaved caspase-1, which leads to the occurrence of COPD airway inflammation. After MgIG intervention treatment, the relative expression of NLRP3 and Cleaved caspase-1 proteins were significantly reduced in LOW and HIGH group as that in SMF group. That implied that MgIG can inhibit airway inflammation in COPD model rats, and its mechanism of action is associated to down-regulation of the NLRP3 signaling pathway.

## Limitations

COPD is a common, preventable, and treatable chronic lung disease, but its pathophysiological mechanisms is complicated. And, the clinical outcome of COPD is associated with complications it caused, such as pulmonary hypertension [43]. Moreover, chronic consumption of glycyrrhizic acid may cause peseudohyperaldosteronism and hypertension due to its inhibition effect on 11-β-hydroxysteroid dehydrogenase type-2 (11βHSD2) [44]. So, the effect of MgIG on COPD needs further systematic study comprising those factors mentioned above, although we did not observe other abnormal appearance of all rats in this study. MgIG has a higher affinity to the liver after oral or injection, and it is mainly used to treat liver diseases in clinical practice. Although the method of administration in this study was pulmonary administration, avoiding the high affinity of the liver to a certain extent, individual differences in lung structure and function in rats would also affect the absorption of the drug. Additionally, state of downstream signal of the NLRP3 signaling pathway needs further evaluation to clear the effect of MgIG on NLRP3 pathway to control COPD.

## Conclusions

The present study demonstrated that MgIG attenuated pathological changes in rats with COPD by inhibiting airway inflammation and the NLRP3 signaling pathway. These findings suggest MgIG might be an alternative for COPD therapy.

## Supplementary Information

**Additional file 1.** The original full-length gels and blots of western blot analysis.

## Data Availability

The datasets used and/or analyzed during the current study are available from the corresponding author on reasonable request.

## Abbreviations

COPD: Chronic obstructive pulmonary disease
BALF: Bronchoalveolar lavage fluid
ETA: Endotracheal-atomization
FVC: The forced vital capacity
FEV0.3: The forced expiratory volume in 0.3 seconds
HE: Hematoxylin and eosin
IL: Interleukin
LPS: Lipopolysaccharide
LTB4: Leukotriene B4
MgIG: Magnesium isoglycyrrhizinate
PMSF: Phenylmethanesulfonyl fluoride
TNF: Tumor necrosis factor
