# Supplementary figures and images for: Magnesium isoglycyrrhizinate inhibits airway inflammation in rats with chronic obstructive pulmonary disease
Source: BMC Pulm Med. 2021 Nov 15;21:371. doi: 10.1186/s12890-021-01745-7 (PMC8590971; doi:10.1186/s12890-021-01745-7)

6A

β-actin


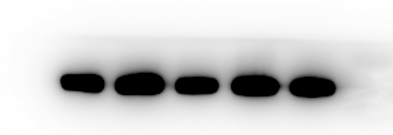


Cleaved caspase-1


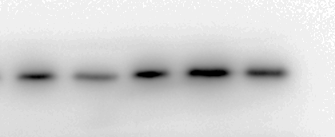


NLRP3


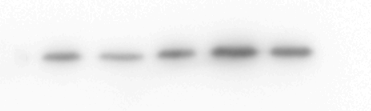

Supplement: Supplementary file 1 — Additional file 1. The original full-length gels and blots of western blot analysis. [file 12890_2021_1745_MOESM1_ESM.docx]
